# *Micromeria fruticosa* Induces Cell Cycle Arrest and Apoptosis in Breast and Colorectal Cancer Cells

**DOI:** 10.3390/ph13060115

**Published:** 2020-06-03

**Authors:** Waseem El-Huneidi, Naglaa G. Shehab, Khuloud Bajbouj, Arya Vinod, Ahmed El-Serafi, Jasmin Shafarin, Lara J. Bou Malhab, Wael M. Abdel-Rahman, Eman Abu-Gharbieh

**Affiliations:** 1 Department of Basic Medical Sciences, College of Medicine, University of Sharjah, Sharjah 27272, UAE; welhuneidi@sharjah.ac.ae (W.E.-H.); kbajbouj@sharjah.ac.ae (K.B.); 2 Department of Pharmaceutical Chemistry and Natural Products, Dubai Pharmacy College, Dubai 19099, UAE; naglaa@dpc.edu; 3 Department of Pharmacognosy, Faculty of Pharmacy, Cairo University, Giza 12613, Egypt; 4 Sharjah Institute for Medical Research, University of Sharjah, Sharjah 27272, UAE; arya.vinodnadat@gmail.com (A.V.); jsalam@sharjah.ac.ae (J.S.); lara.boumalhab@hotmail.com (L.J.B.M.); whassan@sharjah.ac.ae (W.M.A.-R.); 5 Department of Biomedical and Clinical Sciences, Linköping University, SE-581 85 Linköping, Sweden; ahmed.elserafy@liu.se; 6 Medical Biochemistry and Molecular Biology Department, Faculty of Medicine, Suez Canal University, Ismailia 41522, Egypt; 7 Department of Medical Laboratory Sciences, College of Health Sciences, University of Sharjah, Sharjah 27272, UAE; 8 Department of Clinical Sciences, College of Medicine, University of Sharjah, Sharjah 27272, UAE

**Keywords:** *Micromeria fruticosa*, CDK1, cyclin B1, breast cancer, colon cancer

## Abstract

*Micromeria fruticosa* (L.) Druce subsp. *serpyllifolia* (Lamiaceae) has been used widely in folk medicine to alleviate various ailments such as abdominal pains, diarrhea, colds, eye infections, heart disorders and wounds. A few reports have confirmed different therapeutic potentialities of its extracts, including the anti-inflammatory, gastroprotective, analgesic, antiobesity and antidiabetic activities. This study aimed to investigate the mechanistic pathway of the antiproliferative activity of the ethanolic extract of *M. fruticosa* on two different cancer cell lines, namely human breast (mammary carcinoma F7 (MCF-7)) and human colorectal (human colon tumor cells (HCT-116)) cell lines. The 3-(4,5-Dimethylthiazol-2-yl)-2,5-diphenyltetrazolium bromide tetrazolium (MTT) assay, Annexin V-FITC/PI, caspases 8/9 and cell cycle analyses, qRT-PCR and Western blot were used to assess the effect of *M. fruticosa* on cytotoxicity, apoptosis, cell cycle, cell cycle-related genes and protein expression profiles in MCF-7 and HCT-116. The extract inhibits cell proliferation in a time- and dose-dependent manner. The half-maximal inhibitory concentration (IC_50_) for both cell lines was found to be 100 μg/mL. Apoptosis induction was confirmed by Annexin V-FITC/PI, that was related to caspases 8 and 9 activities induction. Furthermore, the cell cycle analysis revealed arrest at G2/M phase. The underlying mechanism involved in the G2/M arrest was found to be associated with the downregulation of CDK1, cyclin B1 and survivin that was confirmed by qRT-PCR and Western blotting.

## 1. Introduction

Globally, cancer is reported to be the second leading cause of death, where it causes one death in every six deaths worldwide [1]. According to the World Health Organization, cancer accounted for 9.6 million deaths in 2018 [1]. Despite the fact that chemotherapy has now progressed towards more targeted therapy against cancer, it is still straggled with many unpreventable complications and side effects [2].

For centuries, medicinal plants have been considered as a treasured source of different types of natural remedies. Although around one-fourth of the prescribed medications in the world originates from wild or cultivated plants [3], only a small percentage of plant species have been investigated for their antineoplastic activities.

*Micromeria fruticosa (L.) Druce subsp. Serpyllifolia* is a perennial aromatic shrub belongs to the *Lamiaceae* family and widely grown in the Mediterranean regions [4]. The plant is commonly used in folk medicine as a herbal infusion to alleviate many illnesses, such as abdominal cramps, diarrhea, colds, ocular infection, cardiac disorders and wounds [5,6]. Few reports on *M. fruticosa* have confirmed its multi-therapeutic potentialities, such as anti-inflammatory, gastroprotective [7,8], analgesic [9], antiobesity and antidiabetic activities [4]. Moreover, the aqueous extract and the volatile oil of *M. fruticosa* were previously screened for their antitumor activities against human colon tumor cells (HCT-116) and mammary carcinoma F7 (MCF-7), however, the underlying mechanism was not explored [10]. The aqueous extract of the plant was also reported to induce the growth inhibition of glioblastoma multiforme cells through the induction of oxidative stress in brain cancer cells [11]. 

The aim of the current study was to investigate the mechanistic pathway for the antitumor activity of the ethanolic extract of *M. fruticosa* on two different cancer cell lines, namely human breast (MCF-7) and human colorectal (HCT-116) cell lines.

## 2. Results

### 2.1. Cytotoxic Activity

**Figure 1:**
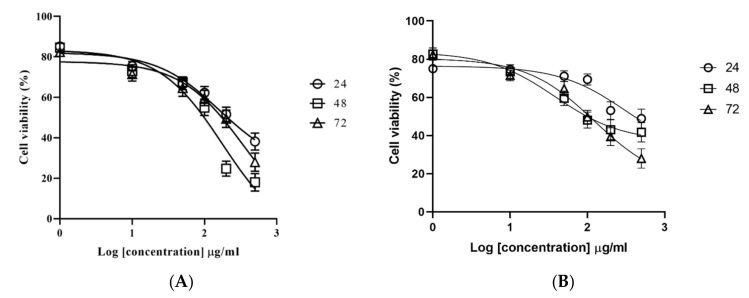
Effects of the *M. fruticosa* extract on the viability of (**A**) the mammary carcinoma F7 (MCF-7) and (**B**) human colon tumor (HCT-116) cells at 24, 48 and 72 h. Data from at least three independent experiments performed in at least triplicates are presented as the means ± SD.

To investigate the effects of *M. fruticosa* extract on cancer cell proliferation, HCT-116 and MCF-7 cells were treated with increasing concentrations of the extract (0, 1, 10, 50, 100, 200 and 500 μg/mL) for 24, 48 and 72 h and the dose–response curve was used to calculate the IC_50_ values. The results showed that the *M. fruticosa* extract decreased the cell viability in a dose- and time-dependent manner in both cell lines (Figure 1) with an IC_50_ of 208.0, 99.17, and 173.8 µg/mL after 24, 48 and 72 h incubation against the MCF-7 cell line, respectively, and an IC_50_ of 403.8, 98.8 and 106.3 µg/mL for 24, 48 and 72 h incubation for the HCT-116 cell line, respectively. Accordingly, the concentration of the *M. fruticosa* extract at 100 µg/mL for 48 h was selected for further analysis.

### 2.2. Apoptotic Activity

Annexin V-FITC/PI double staining assay was used to assess the proapoptotic activity of the extract on both cell lines that were treated with 100 µg/mL of *M. fruticosa* extract for 48 h. 

**Figure 2:**
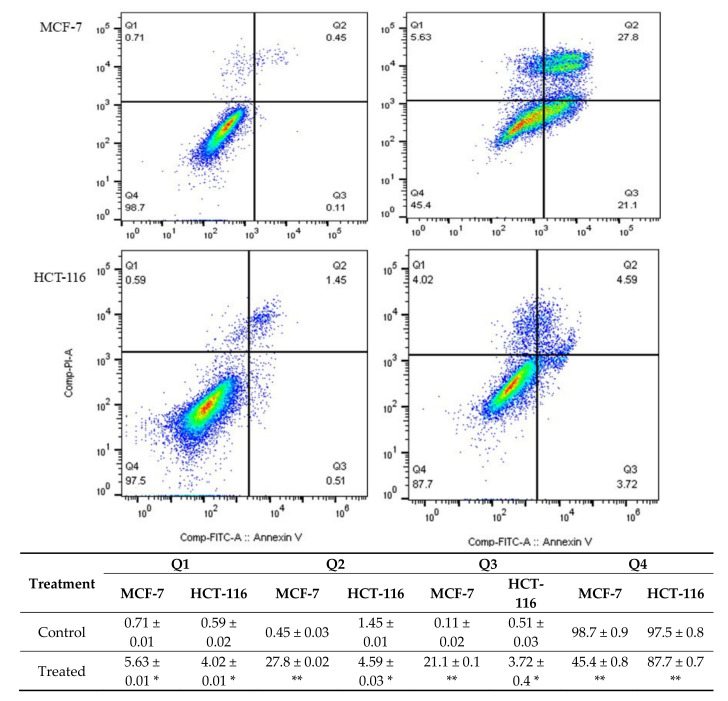
The level of apoptosis and necrosis was assessed by flow cytometry using Annexin V/PI staining, the control groups were without any treatment, and the *M. fruticosa* extract groups were treated with 100 μg/mL *M. fruticosa* extract for 48 h, * *p* < 0.05, ** *p* < 0.001. Q1: necrotic cells, Q2, late apoptotic, Q3: early apoptotic, Q4: living cells.

As shown in Figure 2, the rate of Annexin V-positive revealed that apoptotic cells were significantly increased by treating both cell lines with the *M. fruticosa* extract. The early and late apoptotic rates in the MCF-7 treated cells were 21.1% and 27.8% compared to the control of 0.11% and 0.45%, respectively. On the other hand, the apoptotic rates of the treated HCT-116 cells were also significantly increased, indicating early-stage apoptosis.

### 2.3. Caspase Activity

**Figure 3:**
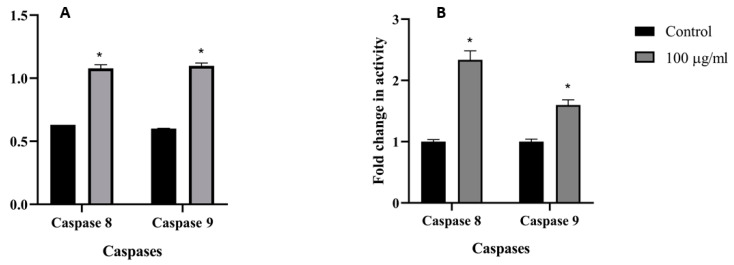
Caspases 8 and 9 activity on the MCF-7 (**A**) and the HCT-116 (**B**) cell lines, the control groups were without any treatment, and the *M. fruticosa* extract groups were treated with 100 µg/mL for 48 h, * *p* < 0.01.

To further assess the *M. fruticosa* extract-induced apoptosis in the MCF-7 and HTC-116 cells, intracellular apoptotic molecular biochemical events were examined. Figure 3 showed that the activities of both caspases 8 and 9 were significantly induced in the cells treated with the *M. fruticosa* extract at 100 µg/mL for 48 h compared to the untreated cells.

### 2.4. Cell Cycle Analysis

**Figure 4:**
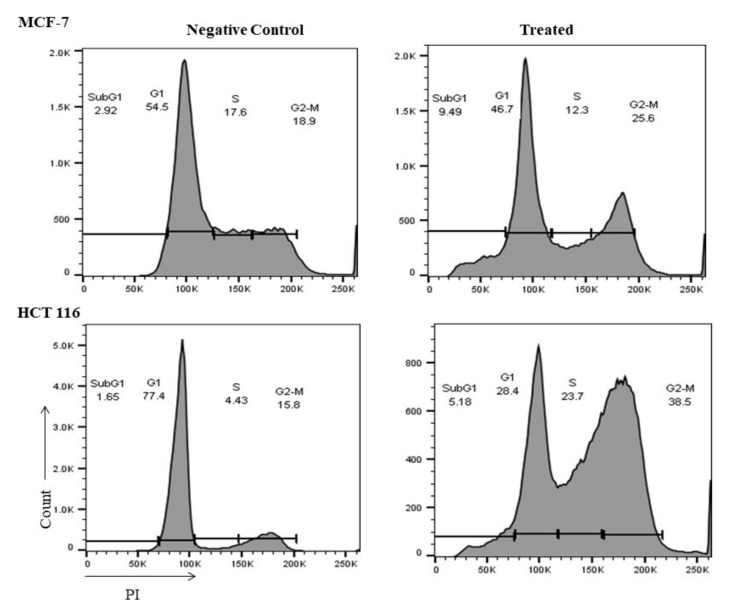
*M. fruticosa* extract induces cell cycle arrest in the MCF-7 and the HCT-116 cells. The cells were treated with DMSO (negative control), and with the *M. fruticosa* extract (100 µg/mL) for 48 h. Flow cytometric analysis was performed for the cell-cycle distribution. The DNA content was evaluated with propidium.

To explore the impact of the *M. fruticosa* extract on the cell cycle progression in both cell lines, the DNA content was measured by flow cytometry. Figure 4 showed that the *M. fruticosa* extract was able to induce cell cycle arrest at G2/M in MCF-7 and HTC-116 cells when compared to their controls, from 18.9% to 25.6% and from 15.8% to 38.5%, respectively. There was a remarkable increase in sub-G1 population in MCF-7 and HTC-116 after *M. fruticosa* extract treatment by 9% and 5%, respectively, which confirmed the apoptosis induction after 48 h.

### 2.5. qRT-PCR and Western Blot

**Figure 5:**
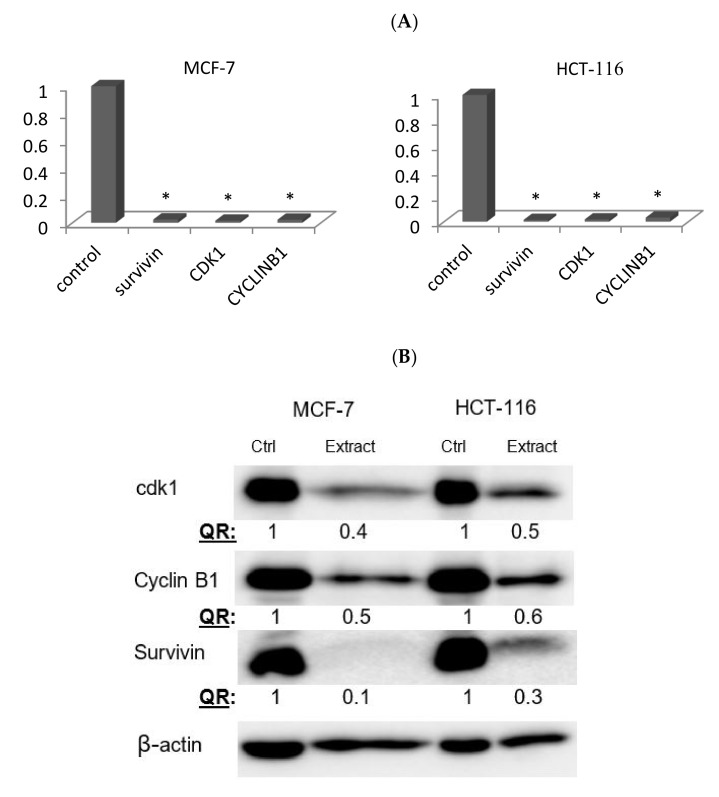
The effect of the *M. fruticosa* extract on the expressions of cdk1, cyclin B1 and survivin in MCF7 and HCT-116 cancer cell lines. Both cells were treated with the vehicle (0.1% DMSO) or treated with the extract at 100 µg/mL for 48 h. (**A**) The relative fold regulation of the survivin, cyclin B1 and cdk1 gene expression by qRT-PCR, * *p* < 0.01. (**B**) The relative protein expression obtained by Western blotting. The numbers represented here indicate the relative protein quantity normalized to β-actin and comparing treated cells to the control counterparts. QR: represents the quantitative analysis of the band density after normalization to the control.

To investigate the molecular mechanisms involved in the apoptotic induction and G2/M arrest, the expression of several genes and proteins regulating cell death (survivin) and G2/M transition (cyclin B and Cdk1) were assessed using qRT-PCR. The expression levels of survivin, cyclin B, and Cdk1 genes were significantly down regulated in both treated cell lines compared to the control counterparts. Similarly, Western blotting further confirmed a significant reduction of survivin, cdk1 and cyclin B proteins by 90%, 60%, 50% in MCF-7, respectively, and by 70%, 50% and 40% and in HCT116, respectively, as shown in Figure 5.

## 3. Discussion

*Micromeria fruticosa* is a Mediterranean medicinal plant that is used to treat different health conditions. The total phenolic and flavonoid contents of the plant’s alcoholic extract were previously reported by the authors [9]. Reversed-phase high-performance liquid chromatography (RP-HPLC) was employed for the identification and quantification of the phenolics of the extract [9]. The extract was found to contain a high phenolic content (3.92 mg/g), of which ferulic acid and rosmarinic acid were predominant (4.30% and 4.23%, respectively), followed by a non-phenolic compound, benzoic acid (2.08%). Additionally, the plant had a high flavonoidal content (1.01%), of which quercitrin was prevalent (4.72%) [9].

The cytotoxicity of the aqueous extract and the hydro-distillate oil of the plant were described earlier by the authors in both breast and colon cancer cells [10]; however, the exact mechanism of action of the observed findings was not explored. Furthermore, the antiproliferative activity of the aqueous extract of the plant was reported against a brain cancer cell line (U-87 MG) that was related to the induction of growth inhibition and oxidative stress [11]. As a continuation of our previous observation on the cytotoxic effect of the *M. fruticosa* extract on breast and colon cancer, the ethanolic extract was selected in this study as ethanol was found to be the best solvent for plant extraction with the highest yield of phenolics and flavonoids compared to other solvents [9]. That said, the present study aimed to examine the potential molecular mechanisms of *M. fruticosa* extract on human breast (MCF-7) and human colorectal (HCT-116) cell lines.

Based on the 3-(4,5-Dimethylthiazol-2-yl)-2,5-diphenyltetrazolium bromide tetrazolium (MTT) assay, it is evident that the cells exposed to the *M. fruticosa* extract lose their ability to proliferate in a time- and dose-dependent manner, which can be partially due to the cell death induction. On both cell lines, a dose of 100 µg/mL with 48 h was chosen for further investigation depending on the calculated IC_50_ with the maximum inhibitory effect.

To investigate if the cell death caused by *M. fruticosa* extract was induced by apoptotic activity, the Annexin V-FITC/PI assay was used. This technique monitors the translocation of phosphatidylserine from the inner side of the cell membrane to the outer layer to detect early apoptosis [12]. The quantification of apoptotic cells was gained by measuring the Annexin V-FITC that binds to the externalized phosphatidylserine. Propidium iodide (PI) was added to differentiate between viable, early apoptotic, late apoptotic and necrotic cells [13]. The analysis of MCF-7 and HCT-116 cells by flow cytometry revealed that treatment with the *M. fruticosa* extract induced a shift in the cell population toward apoptosis. After treating the MCF-7 cells with 100 µg/mL of the *M. fruticosa* extract for 48 h, the early and late apoptotic rates were significantly increased compared to the control group (*p* < 0.001). Similarly, a significant change in the apoptotic stages was observed with HCT-116 (*p* < 0.05), as shown in Figure 2. These results demonstrate the ability of *M. fruticosa* extract to induce apoptosis, particularly in MCF-7 cells.

Caspases are essential proteases that control the apoptotic pathway [14]. Caspase activation analysis in this study was performed on caspases 8 and 9 since they are present in both cell lines. Caspase 3 was not selected because the MCF-7 cell line, used in this study, was established from a Caucasian woman breast cancer which lacked caspase 3 activity because of a point mutation in the gene which encodes caspase 3 [15,16]. The findings showed that the *M. fruticosa* extract activated caspase 8 and 9 on the tested cell lines, which indicated that the apoptotic effect of the extract depends on both caspase 8 activity, representing the extrinsic cell death pathway, and caspase 9 that is associated with the intrinsic cell death pathway. The cross-communication could explain the remarked activation of caspase 9 between the intrinsic and extrinsic pathways mediated by the cleavage of the proapoptotic Bid (Bcl-2 family member) [17,18].

The significant proapoptotic activity of the extract on the tested cell lines could be allied to the high content of many bioactive molecules, including ferulic, rosmarinic and benzoic acids and quercitrin. Phenolic and flavonoid compounds are among the most important plants’ secondary metabolites, as they possess many biological properties due to their molecular structure, including antioxidant activity [19].

Ferulic acid, as the major phenolic acid in the plant extract, is reported for its proapoptotic and antiproliferative activities against various cancer cell lines [20,21]. Rosmarinic acid was also reported to induce cell cycle arrest and apoptosis on many cancer cell lines [22,23]. In prostate cancer, it induced apoptosis and cell cycle arrest through the modulation of HDAC2 expression [24]. Moreover, benzoic acid showed significant cytotoxic and antiproliferative activities on breast cancer [25]. Quercitrin is a flavonoid with differently known biological activities such as anti-cancer, anti-inflammatory and anti-oxidant [26]. Studies showed evidence that quercetin mediated its anti-cancer activity through proapoptosis and growth inhibition in different cancer cells [27,28,29,30].

With such combinations of various bioactive molecules, synergistic actions against cancer cells were expected.

Cell cycle analysis was conducted to further elaborate on the molecular mechanisms of the noticed cytotoxicity and proapoptotic activity. Results of cell cycle analysis revealed the ability of the extract to arrest cell cycle on both cell lines at G_2_/M phase. The cell cycle is mainly regulated through checkpoints that ensure the accurate progress of the cells over the cell cycle. The G2/M checkpoint, specifically, inhibits the entry into mitosis phase when DNA is damaged [31]. Accordingly, the expression of the three genes and their related proteins, namely survivin, cyclin B1 and Cdk1, were studied based on their contribution in cell cycle arrest at the G2/M phase.

Survivin is a protein with an essential role in regulating cell proliferation and apoptotic inhibition. It is not expressed in normal differentiated tissues but upregulated in most tumor tissues [32]. The overexpression of survivin in G2/M phase is usually associated with apoptosis inhibition, the stimulation of cell proliferation and angiogenesis [33]. Moreover, in the premitotic phase, survivin binds with spindle microtubules which causes tumor cells to escape monitoring at the G_2_/M phase of the cell cycle [34]. Cyclin B1, a key player in the cell cycle transition from G2 to M phase, has been known for its role in tumorigenesis and malignancy development. Additionally, cyclin-dependent kinase 1 (Cdk1), formerly known as Cdc2, forms the Cdk1/cyclin B1 complex upon interaction with cyclin B1, and this complex regulates the transition from G2 to M phase [35].

Results from the qt-PCR and Western blot confirmed the significant downregulation effect of the *M. fruticosa* extract on the expression of survivin, cyclin B and Cdk1 mRNAs, as well as their corresponding proteins, which in sum explains the observed cell cycle arrest along with the proapoptotic activity of the extract on the tested cell lines.

## 4. Materials and Methods

### 4.1. Chemicals

Ethanol, Dimethyl sulfoxide (DMSO) and 3-(4,5-Dimethylthiazol-2-yl)-2,5-diphenyltetrazolium bromide tetrazolium (MTT) were purchased from Sigma-Aldrich (Saint Louis, MO, USA). The kits for caspase 8 (ab39700) and 9 ELISA (ab65608), Annexin V-FITC (ab14085) and PI staining (ab139418) kits were purchased from Abcam, USA. The RNeasy Mini Kit (74104) and the cDNA synthesis kit (54420) were purchased from Qiagen, Germany and Norgen Biotek, Canada, respectively. All solvents were of analytical grade and obtained from Fisher Scientific (UK).

### 4.2. Plant Material and Extraction

The aerial parts of *M. fruticosa* were collected in July 2015 from Nablus, Palestine. The plant was kindly authenticated by Prof. Hassnaa Hosny, Cairo University, Egypt. A plant sample was kept at Dubai Pharmacy College Herbarium (# 8-7-15). The air-dried plant was powdered and held for extraction.

The powdered plant (700 g) was extracted exhaustively by cold maceration in ethanol (3 L 2). The alcohol was evaporated using a rotary evaporator at 50 °C, giving 100 g residue.

### 4.3. Cell Lines

The human breast (MCF-7) and human colorectal (HCT-116) cell lines were obtained from the American Type Culture Collection (ATCC). The cells were seeded in T-75 culture flasks (Thermo Scientific, Waltham, MA, USA) and maintained in Dulbecco’s modified Eagle‘s medium (DMEM) media, supplemented with 10% fetal bovine serum (FBS), 100 U/mL streptomycin and 100 U/mL penicillin. The plates were kept at 37 °C with 5% carbon dioxide. Every two days, the medium was changed, and the cells were passaged when they reached about 80% confluence.

### 4.4. Cytotoxicity Assay

A stock solution of the plant extract was prepared by dissolving 10 mg of the extract in 1 mL of DMSO, and then used to prepare a range of concentration from 1 to 500 μg/mL. The cells were plated in a 96-well plate (5 × 10^3^ cells/well) and incubated with the extracts for 24, 48 and 72 h in triplicates. Following treatment, the MTT stock solution (5 mg/mL in phosphate-buffered saline, PBS) was added to the medium, and the cells were incubated for 2 h at 37 °C to allow for the cell-mediated reduction of MTT. A hundred microliters of DMSO were added to solubilize the MTT crystals followed by an incubation of 10 min. The absorbance was read at 570 nm in a microtiter plate reader, and the proliferation rate was measured by comparing the absorbance of extract-treated cultures with the untreated controlled cultures.

### 4.5. Annexin V/PI

The annexin-V-PI staining method was used to detect the apoptosis induction. Briefly, following the Annexin V-FITC Detection Kit protocol (Abcam, Cambridge, MA, USA), both cell types were incubated with the IC_50_ value of the extract (100 µg/mL) for 48 h. The cells were then harvested, washed twice with PBS and stained for 20 min with staining buffer containing annexin-V/PI. The cells were afterwards analyzed for apoptosis by using Flow cytometer (BD FACS Aria III; Becton Dickinson) at 488 nm excitation; a 530/30 nm bandpass filter for fluorescein detection and a long pass filter 502 nm were used. PI-positive cells were only considered to be necrotic; cells positive for annexin V staining were only counted as early apoptotic; while cells positive for annexin V and PI were considered to be at the late apoptotic stage. Flow cytometry data were analyzed using the *FlowJo* software with Watson pragmatic model (Tree Star, Ashland, OR, USA).

### 4.6. Caspase 8/9 Assay

Following the manufacturer’s protocol, briefly, MCF-7 and HCT-116 cells were incubated with the extract at a concentration of 100 µg/mL for 48 h, followed by mechanical disruption. After repeated centrifugation and vortexing, the total protein was isolated and then quantitated using nanodrop. Fifty micrograms of the total protein were utilized for the estimation of Caspase 8/9, and the absorbance was measured at 400 nm. The percentage of activity was calculated by comparing the absorbance of the extract-treated cells with the untreated cells.

### 4.7. Cell Cycle Analysis

Flow cytometry was used to analyze the distribution of the cell cycle phases. Briefly, the MCF-7 and HCT-116 cells were seeded at a density of 0.5 × 10^6^ cells and treated with 100 µg/mL of the *M. fruticosa* extract for 48 h. After that, cells were harvested and fixed using 1 mL of 70% ethanol at −20 °C overnight. The cells were washed with PBS, stained with PI and DNase-free RNase solution for 30 min. The cells and their progression through different cell cycle phases were then analyzed using flow cytometry platform.

### 4.8. RNA Isolation, cDNA and qRT-PCR

The total RNA was extracted from both the treated and untreated MCF-7 and HCT-116 cells, using the RNeasy Mini Kit (Qiagen, Hilden, Germany), according to the manufacturer’s instruction. The NanoDrop 2000 spectrophotometer (Thermo Scientific, Waltham, MA, USA) was used to determine the RNA concentration. One microgram of the total RNA was used for the synthesis of cDNA, based on the manufacturer’s protocol. For qRT-PCR, SsoFast EvaGreen Supermix (Bio-Rad, CA, USA) was used and all the reactions were processed in the Bio-rad CFX-96 real-time PCR instrument. Then, 18S ribosomal RNA (18S rRNA) was used as the reference gene. The primers used for the target gene amplification are listed in Table 1. The 2^−ΔΔCt^ method was used to calculate the fold change.

### 4.9. Western Blotting Analysis

The MCF-7 and HCT-116 cells were lysed in ice-cold NP40 lysis buffer containing protease cocktail-inhibitor (Sigma-Aldrich). The protein concentrations were calculated using the Bradford method (Bio-Rad). Then, 30 μg protein were loaded and separated by 12% SDS-PAGE and then blotted onto a nitrocellulose membrane (Bio-Rad). The membrane was then blocked with 5% skimmed milk powder, then washed with Tris-buffered saline, 0.1% Tween 20 (TBST), and incubated at 4 °C overnight with primary IgG-unlabeled antibodies, anti-survivin (EPR2675) (ab134170); anti-CDK1 (A17) (ab18) and anti–cyclinB (Y106) (ab32053), purchased from Abcam, Cambridge, MA, USA. Secondary antibodies (antimouse and antirabbit; Cell Signaling Technology) were reacted for one hour with the membrane at 1:1000 dilutions. Chemiluminescence (Thermo Fisher Scientific) was used to detect the bands and Image Lab software (ChemiDoc Touch Gel and Western blot imaging system; Bio-Rad) was used to quantify the band-density; β-actin was used as a normalization control.

### 4.10. Statistical Analysis

All the experiments were performed in triplicates and the data were expressed as the mean ± standard deviation (SD). GraphPad Prism 8 was for the statistical analysis. Differences between the means were analyzed by a Student’s *t*-test, and *p* < 0.05 was considered statistically significant.

## 5. Conclusions

The ethanolic extract of *Micromeria fruticosa* induced apoptosis and cell cycle arrest in breast and colon cancer cell lines. Mechanistic studies revealed that the extract induced the expression of caspases 8 and 9, and targeted the G2/M phase by effecting the expression of the different proteins involved in this phase. The findings of this study provided a ground base to initiate in vivo studies to evaluate the efficacy of *M. fruticosa* extract on animal models as a complement to the currently used chemotherapies against breast and colon cancers.

## Figures and Tables

**Table 1:** List of primers used for the quantitative real-time PCR.

Genes	Forward (5′→3′)	Reverse (5′→3′)
Survivin	5’ACCGCATCTCTACATTCAAG3’	5’CAAGTCTGGCTCGTTCTC3′
CDK1 Cyclin B1	5’CAGACTAGAAAGTGAAGAGGAAGG3’ 5’AAGAGCTTTAAACTTTGGTCTGGG3’	5’ACTGACCAGGAGGGATAGAA3′ 5’GTTTGTAAGTCCTTGATTTACCATG3′
18S rRNA	5’TCAGATACCGTCGTAGTTCCG3’	5’CAGCTTTGCAACCATACTCCC3′

## References

[1] World Health Organization (1951). Cancer registration and statistics. J. Am. Med. Womens Assoc..

[2] MacDonald V. (2009). Chemotherapy: Managing side effects and safe handling. Can. Vet. J..

[3] Zi X., Zhang R. (2013). Anti-cancer molecular targets of natural products. Curr. Cancer Drug Targets.

[4] Salameh N., Shraim N., Jaradat N. (2018). Chemical Composition and Enzymatic Screening of Micromeria fruticosa serpyllifolia Volatile Oils Collected from Three Different Regions of West Bank, Palestine. Biomed. Res. Int..

[5] Yaniv Z., Dudai N. (2014). Medicinal and Aromatic Plants of the Middle-East.

[6] Dafni A., Yaniv Z., Palevitch D. (1984). Ethnobotanical survey of medicinal plants in northern Israel. J. Ethnopharmacol..

[7] Abu-Gharbieh E., Shehab N.G., Khan S.A. (2013). Anti-inflammatory and gastroprotective activities of the aqueous extract of *Micromeria fruticosa* (L.) Druce ssp Serpyllifolia in mice. Pak. J. Pharm. Sci..

[8] Abu-Gharbieh E., Bustanji Y., Mohammad M. (2010). In vitro effects of *Micromeria fruticosa* on human leukocyte myeloperoxidase activity. J. Pharm. Res..

[9] Abu-Gharbieh E., Ahmed N.G. (2016). Bioactive content, hepatoprotective and antioxidant activities of whole plant extract of *Micromeria fruticosa* (L) Druce ssp *Serpyllifolia* F Lamiaceae against Carbon tetrachloride-induced hepatotoxicity in mice. Trop. J. Pharm. Res..

[10] Shehab N.G., Abu-Gharbieh E. (2012). Constituents and biological activity of the essential oil and the aqueous extract of *Micromeria fruticosa* (L.) Druce subsp. serpyllifolia. Pak. J. Pharm. Sci..

[11] Koc K., Ozdemir O., Kizilkaya O.F., Sengul M., Turkez H. (2017). Cytotoxic activity of the aqueous extract of *Micromeria fruticosa* (L.) Druce subsp. serpyllifolia on human U-87 MG cell lines. Arch. Biol. Sci..

[12] Liu T., Zhu W., Yang X., Chen L., Yang R., Hua Z., Li G. (2009). Detection of apoptosis based on the interaction between annexin V and phosphatidylserine. Anal. Chem..

[13] Baskic D., Popovic S., Ristic P., Arsenijevic N.N. (2006). Analysis of cycloheximide-induced apoptosis in human leukocytes: Fluorescence microscopy using annexin V/propidium iodide versus acridin orange/ethidium bromide. Cell Biol. Int..

[14] Julien O., Wells J.A. (2017). Caspases and their substrates. Cell Death Differ..

[15] Janicke R.U., Sprengart M.L., Wati M.R., Porter A.G. (1998). Caspase-3 is required for DNA fragmentation and morphological changes associated with apoptosis. J. Biol. Chem..

[16] Srinivasula S.M., Ahmad M., Fernandes-Alnemri T., Litwack G., Alnemri E.S. (1996). Molecular ordering of the Fas-apoptotic pathway: The Fas/APO-1 protease Mch5 is a CrmA-inhibitable protease that activates multiple Ced-3/ICE-like cysteine proteases. Proc. Natl. Acad. Sci. USA.

[17] Ashkenazi A. (2008). Targeting the extrinsic apoptosis pathway in cancer. Cytokine Growth Factor Rev..

[18] Jelinek M., Balusikova K., Schmiedlova M., Nemcova-Furstova V., Sramek J., Stancikova J., Zanardi I., Ojima I., Kovar J. (2015). The role of individual caspases in cell death induction by taxanes in breast cancer cells. Cancer Cell Int..

[19] Dai J., Mumper R.J. (2010). Plant phenolics: Extraction, analysis and their antioxidant and anticancer properties. Molecules.

[20] ElKhazendar M., Chalak J., El-Huneidi W., Vinod A., Abdel-Rahman W.M., Abu-Gharbieh E. (2019). Antiproliferative and proapoptotic activities of ferulic acid in breast and liver cancer cell lines. Trop. J. Pharm. Res..

[21] Zhang X.D., Wu Q., Yang S.H. (2017). Ferulic acid promoting apoptosis in human osteosarcoma cell lines. Pak. J. Med. Sci..

[22] Han Y.H., Kee J.Y., Hong S.H. (2018). Rosmarinic Acid Activates AMPK to Inhibit Metastasis of Colorectal Cancer. Front. Pharmacol..

[23] Zhang Y., Hu M., Liu L., Cheng X.L., Cai J., Zhou J., Wang T. (2018). Anticancer effects of Rosmarinic acid in OVCAR-3 ovarian cancer cells are mediated via induction of apoptosis, suppression of cell migration and modulation of lncRNA MALAT-1 expression. J. BUON.

[24] Jang Y.G., Hwang K.A., Choi K.C. (2018). Rosmarinic Acid, a Component of Rosemary Tea, Induced the Cell Cycle Arrest and Apoptosis through Modulation of HDAC2 Expression in Prostate Cancer Cell Lines. Nutrients.

[25] Lee K.H., Ho W.Y., Wu S.J., Cheng T.L., Huang P.J., Wang C.C., Hung J.H. (2014). Behavior-selective apoptotic capacity of 4-(3,4,5-Trimethoxyphenoxy) benzoic acid and its methyl derivatives on two breast cancer cell lines. Anticancer Res..

[26] Nguyen L.T., Lee Y.H., Sharma A.R., Park J.B., Jagga S., Sharma G., Lee S.S., Nam J.S. (2017). Quercetin induces apoptosis and cell cycle arrest in triple-negative breast cancer cells through modulation of Foxo3a activity. Korean J. Physiol. Pharmacol..

[27] Bishayee K., Ghosh S., Mukherjee A., Sadhukhan R., Mondal J., Khuda-Bukhsh A.R. (2013). Quercetin induces cytochrome-c release and ROS accumulation to promote apoptosis and arrest the cell cycle in G2/M, in cervical carcinoma: Signal cascade and drug-DNA interaction. Cell Prolif..

[28] Kim G.T., Lee S.H., Kim J.I., Kim Y.M. (2014). Quercetin regulates the sestrin 2-AMPK-p38 MAPK signaling pathway and induces apoptosis by increasing the generation of intracellular ROS in a p53-independent manner. Int. J. Mol. Med..

[29] Maurya A.K., Vinayak M. (2015). Anticarcinogenic action of quercetin by downregulation of phosphatidylinositol 3-kinase (PI3K) and protein kinase C (PKC) via induction of p53 in hepatocellular carcinoma (HepG2) cell line. Mol. Biol. Rep..

[30] Vidya Priyadarsini R., Senthil Murugan R., Maitreyi S., Ramalingam K., Karunagaran D., Nagini S. (2010). The flavonoid quercetin induces cell cycle arrest and mitochondria-mediated apoptosis in human cervical cancer (HeLa) cells through p53 induction and NF-kappaB inhibition. Eur. J. Pharmacol..

[31] Taylor W.R., Stark G.R. (2001). Regulation of the G2/M transition by p53. Oncogene.

[32] Garg H., Suri P., Gupta J.C., Talwar G.P., Dubey S. (2016). Survivin: A unique target for tumor therapy. Cancer Cell Int..

[33] Nestal de Moraes G., Silva K.L., Vasconcelos F.C., Maia R.C. (2011). Survivin overexpression correlates with an apoptosis-resistant phenotype in chronic myeloid leukemia cells. Oncol. Rep..

[34] Li F., Ambrosini G., Chu E.Y., Plescia J., Tognin S., Marchisio P.C., Altieri D.C. (1998). Control of apoptosis and mitotic spindle checkpoint by survivin. Nature.

[35] Norbury C., Nurse P. (1992). Animal cell cycles and their control. Annu. Rev. Biochem..

